# Inductive line tunneling FET using epitaxial tunnel layer with Ge-source and charge enhancement insulation

**DOI:** 10.1186/s11671-023-03878-6

**Published:** 2023-08-05

**Authors:** Jyi-Tsong Lin, Yen-Chen Chang

**Affiliations:** Department of Electrical Engineering, National Sun Yat-Sen University, Kaohsiung, 80424 Taiwan, ROC

**Keywords:** Tunneling effect, Subthreshold swing, Schottky contact, Fermi level pinning, Line tunneling

## Abstract

In this paper, we propose an inductive line tunneling FET using Epitaxial Tunnel Layer with Ge-Source and Charge Enhancement Insulation (CEI ETL GS-iTFET). The CEI ETL GS-iTFET allows full overlap between the gate and source regions, thereby enhancing the line tunneling. In addition, a germanium layer is introduced on the source side to form a heterojunction, effectively improving the device's conduction current. An ETL is incorporated to combat point tunneling leakage, resulting in a steeper subthreshold swing. Furthermore, a CEI consisting of Si_3_N_4_ is introduced between the germanium source and the Schottky metal, which effectively reduces carrier losses in the inversion layer and improves the overall device performance. This study presents a calibration-based approach to simulations, taking into account practical process considerations. Simulation results show that at *V*_D_ = 0.2 V, the CEI ETL GS-iTFET achieves an average subthreshold swing (*SS*_avg_) of 30.5 mV/dec, an* I*_on_ of 3.12 × 10^–5^ A/μm and an *I*_on_/*I*_off_ ratio of 1.81 × 10^10^. These results demonstrate a significantly low subthreshold swing and a high current ratio of about 10^10^. In addition, the proposed device eliminates the need for multiple implantation processes, resulting in significant manufacturing cost reductions. As a result, the CEI ETL GS-iTFET shows remarkable potential in future low-power device competition.

## Introduction

Due to the Boltzmann limit, the subthreshold swing (*SS*) of a MOSFET cannot be lower than 60 mV/dec at room temperature [1]. To overcome the limit, a quantum tunneling mechanism is required [2]. A tunneling field-effect transistor (TFET) achieves a *SS* below 60 mV/dec by utilizing the band-to-band tunneling (BTBT) effect. TFET has several advantages such as low subthreshold swing, low power consumption, and low leakage current. For low-power applications and fast switching, TFET is a promising candidate to replace MOSFET. The structure of a conventional TFET is similar to that of a MOSFET, except for the doping methods used for the source and drain. The doping method for a NMOSFET is N-P-N, while that for a NTFET is P-I-N [3]. The conventional manufacturing process of TFET involves ion implantation and requires high-temperature annealing after implantation. Therefore, the process faces challenges such as increased thermal budget and difficulty in controlling the doping distribution. Furthermore, since conventional Gated PIN/NIP TFET primarily relies on point tunneling for conduction, it faces lots of issues, such as low on-current, gate-dependent *SS* increasing, Trap Assisted Tunneling (TAT), high thermal budget, and ambipolar phenomenon as well, these known issues for the conventional TFET have led to TFET being questioned in the International Roadmap For Devices And Systems™ (IRDS) in 2018 [4]. To overcome the limitations of conventional TFET, researchers have proposed various TFET structures, including Gate-All-Around (GAA) [5], multi-gate [6], heterojunction [7, 8], and other advanced TFET structures. However, ion implantation is still required, which increases production costs and complexity. To address these issues, an inductive line-tunneling dominated iTFET is designed in this work. First, the body is doped with phosphorus. Then, a Schottky contact is used to induce a thin P-type region at the source (*Φ*_m_ > *Φ*_s_) [9], and an Ohmic contact (*Φ*_m_ < *Φ*_s_) is used at the drain to form a Gated PN structure with the gate and the source overlapped. This avoids the need for ion implantation, greatly reducing production costs and complexity.

However, this structure still suffers from low-on current and Fermi level pinning (FLP) issues. The former can be addressed by increasing the overlap area between the source and gate to increase Linear tunneling area [10, 11], and using heterojunctions with additional Germanium material deposited as the source region and exploiting Silicon material as the Epitaxial Tunneling Layer (ETL) for the bulk and drain regions which allow to reduce leakage current and increase drive current as well. Also, to alleviate the impact of FLP and suppress the occurrence of Metal-Induced Gap States (MIGS), a thin insulating layer is inserted at the metal–semiconductor interface [12–15]. This also helps to reduce the loss of minority carrier holes, thereby improving the efficiency and stability of the device. As germanium is a semiconductor with a narrow bandgap and its conduction and valence bands are very close, placing the source and drain regions too close together may result in direct tunneling [16]. Therefore, we modulate the gap height (*h*_gap_) between the source and the top and the length (*L*_D_) of the drain metal to achieve the optimal distance between the source and drain regions, which helps to suppress the ambipolar current [17–19]. Our proposed CEI ETL GS-iTFET is promising for application in TFT displays, as it can reduce power consumption and heat dissipation issues. Additionally, the low sub-threshold swing of the CEI ETL GS-iTFET can enable faster switching, thereby improving the response time of displays, and it has great potential to drive future TFT development. Of course, the CEI ETL GS-iTFET is not limited to display applications. It can also be applied to future electronic devices such as laptops, smartphones, and tablets to meet the demand for high efficiency, high performance, and low power consumption.

## Device structure and simulation framework

**Fig. 1.**
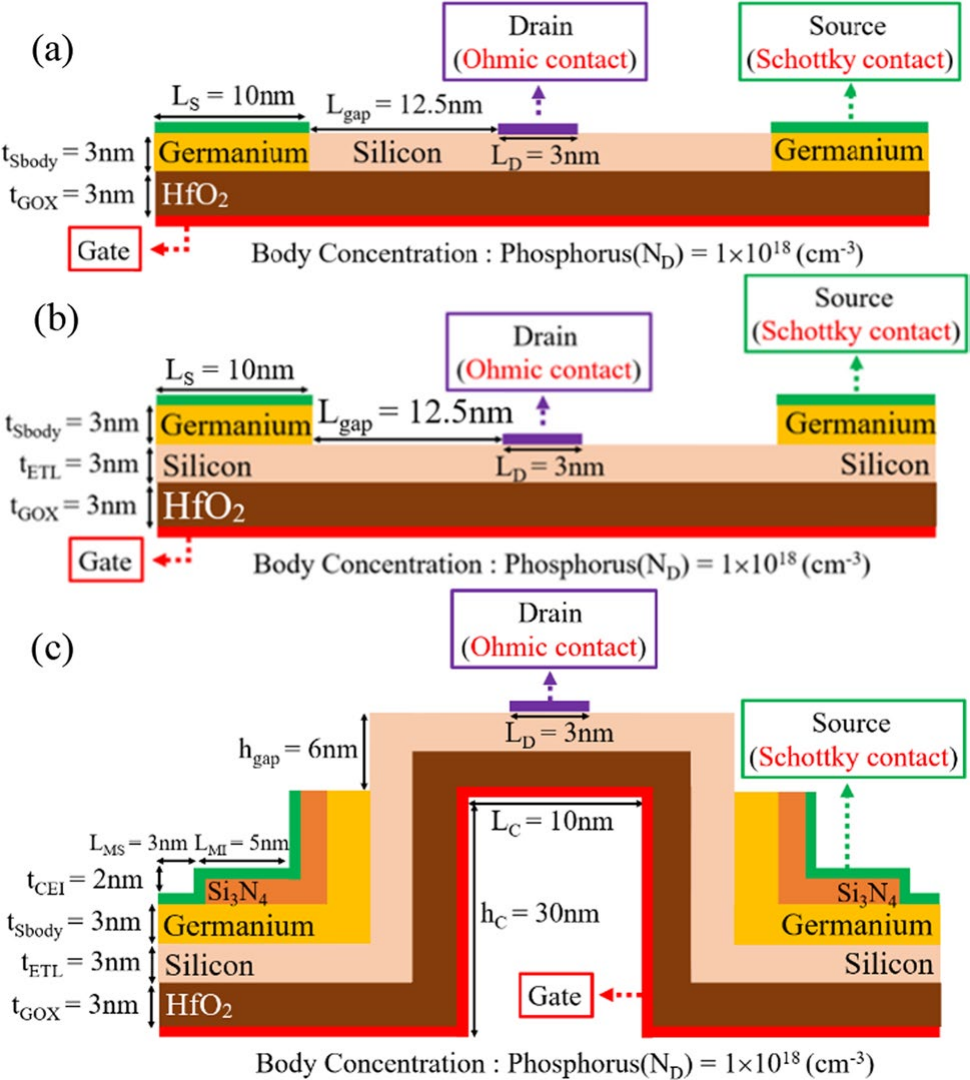
2D schematic of **a** GS-iTFET, **b** ETL GS-iTFET, and **c** CEI ETL GS-iTFET are shown

**Fig. 2:**
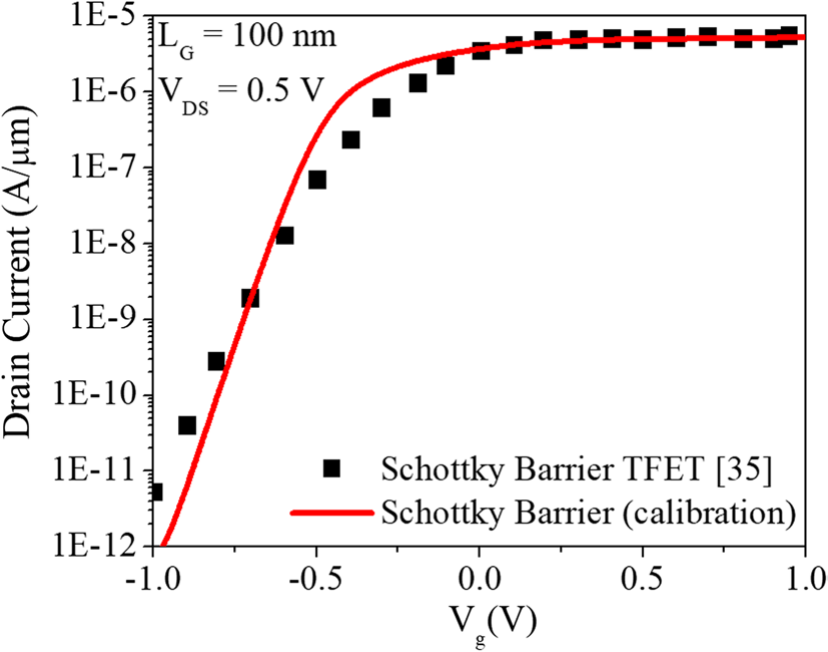
Calibration plot of simulation parameters for the SBTFET

The schematic diagrams of the GS-iTFET with and without ETL and CEI are shown in Fig. 1a–c, respectively. The design and simulation of the device were performed using the Sentaurus TCAD device simulator [20]. The 2D simulator employs various models such as the nonlocal band-to-band tunneling model (BTBT), Hurkx trap-assisted tunneling model (TAT), Shockley–Read–Hall (SRH) recombination model, Auger recombination, Bandgap narrowing (BGN), Fermi level pinning (FLP), Quantum Confinement Effect (QCE), Density-gradient quantization, and Mobility Model to perform simulations. Figure 2 shows the calibration of the simulated model with the actual process results. The Kane's non-local BTB tunneling model was used with parameters for Si (A = 4 × 10^14^ cm^−3^ s^−1^, B = 1.9 × 10^7^ V cm^−1^) [41]. The parameters of the CEI ETL GS-iTFET are shown in Table 1.

**Table 1:** Device parameters used for simulations

Symbol	Value
Source body thickness (*t*_Sbody_)	3 nm
Epitaxial tunnel layer thickness (*t*_ETL_)	3 nm
Gate oxide thickness (*t*_GOX_)	3 nm
CEI thickness (*t*_CEI_)	2 nm
Convex length (*L*_c_)	10 nm
Convex height (*h*_c_)	30 nm
Drain metal length (*L*_D_)	3 nm
Gap height (*h*_gap_)	6 nm
Body doping concentration (phosphorus)	1 × 10^18^ cm^−3^
Schottky barrier	0.7 eV
Gate metal work function	4.1 eV

**Fig. 3:**
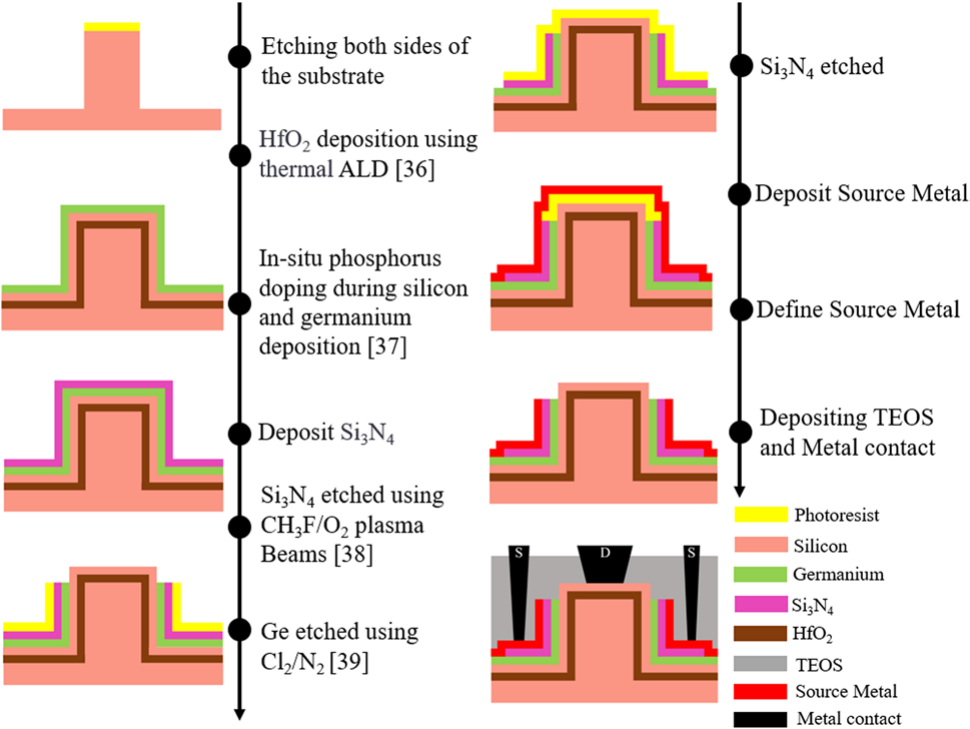
Flowchart of the CEI ETL GS-iTFET manufacturing process

This paper is a simulation work and Fig. 3 shows the assumed manufacturing process of our designed CEI ETL GS-iTFET.

(a) A polycrystalline silicon substrate is used as the back gate electrode. (b) A negative photoresist is used to define the fin shaped structure, followed by dry etching to remove the Polycrystalline silicon. (c) A 3 nm layer of hafnium dioxide is deposited on the polycrystalline silicon as the gate oxide using thermal atomic layer deposition. (d) Silicon and germanium materials are in-situ doped for deposition by high temperature phosphorus evaporation. (e) Low pressure chemical vapor deposition (LPCVD) is used with SiH_2_Cl_2_/DCS and NH_3_ as precursor gases to grow a silicon nitride thin film. (f) Plasma etching with CH_3_F/O_2_ is used to etch the nitride and physical etching with Cl_2_/N_2_ is used to define the drain region. (g) Positive photoresist is used to define the areas where the metal contacts directly contact the semiconductor. (h) Lift-off technique is used to define the source metal. (i) A protective layer of TEOS is deposited by plasma enhanced chemical vapor deposition (PECVD). (j) Finally, metal interconnects are formed by defining contact windows using a photomask, allowing bias to be applied using probes for measurement purposes.

**Fig. 4:**
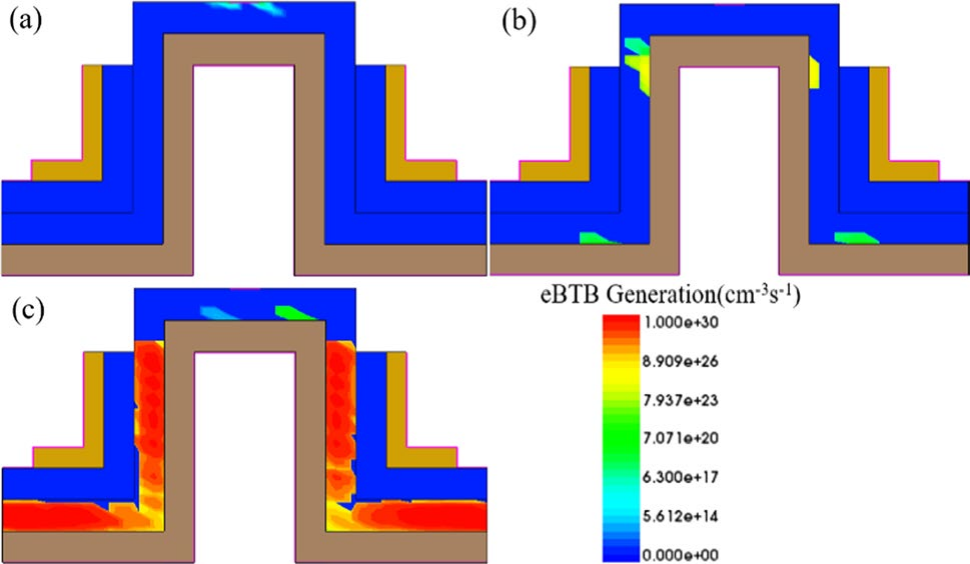
Electron band-to-band generation of the CEI ETL GS-iTFET. **a** Ambipolar conduction, **b** OFF-state, and **c** ON-state

The electron generation rate caused by BTBT in CEI ETL GS-iTFET varies under three different gate bias conditions: (a) ambipolar, (b) OFF-state, and (c) ON-state, with *V*_d_ set at 0.2 V, as illustrated in Fig. 4. At *V*_gs_ = − 1 V, Fig. 4a shows a negligible ambipolar current caused by a slight parasitic tunneling junction. At *V*_gs_ = 0 V, Fig. 4b exhibits a minor corner-point tunneling that has little impact on the overall subthreshold swing. However, in Fig. 4c at *V*_gs_ = 1 V, the electron generation rate fills the entire channel, resulting in a powerful tunneling current.

**Fig. 5:**
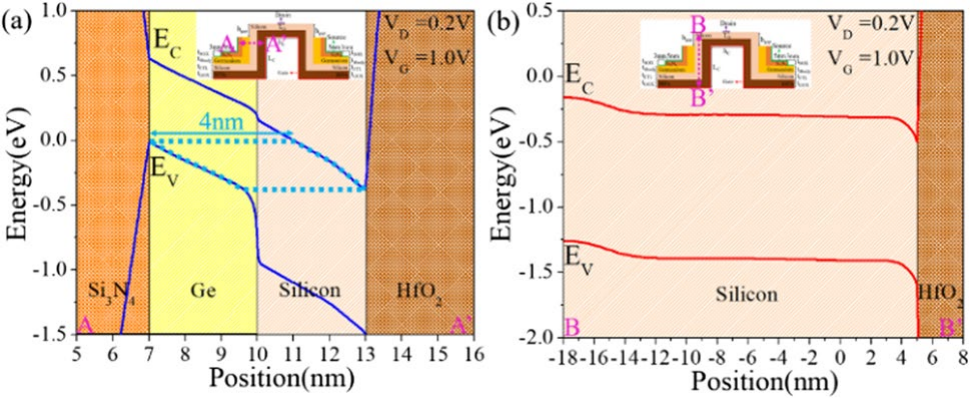
Band diagram of CEI ETL GS-iTFET for **a** line tunneling (along the cut line A–A') and **b** point tunneling (along the cut line B–B')

In traditional TFETs, the current is generated through point tunneling, occurring when the tunneling direction is perpendi-cular to the electric field direction at the tunneling point (along the cut line B–B' in Fig. 5b). In contrast, our proposed CEI ETL GS-iTFET generates tunneling current through line tunneling, where the tunneling direction is parallel to the electric field direction (along the cut line A–A' in Fig. 5a). To maximize the number of line tunneling events, we aim to overlap the source and gate regions as much as possible, rather than relying solely on point tunneling. This allows line tunneling dominant TFETs to have higher drive currents than point tunneling dominant TFETs. Figure 5a clearly shows the overlap region of the conduction and valence bands (light blue area), while Fig. 5b does not have such an overlap region. Therefore, we conclude that the CEI ETL GS-iTFET is dominated by line tunneling.

## Results and discussion

### Optimizing the materials of source and channel regions

**Fig. 6:**
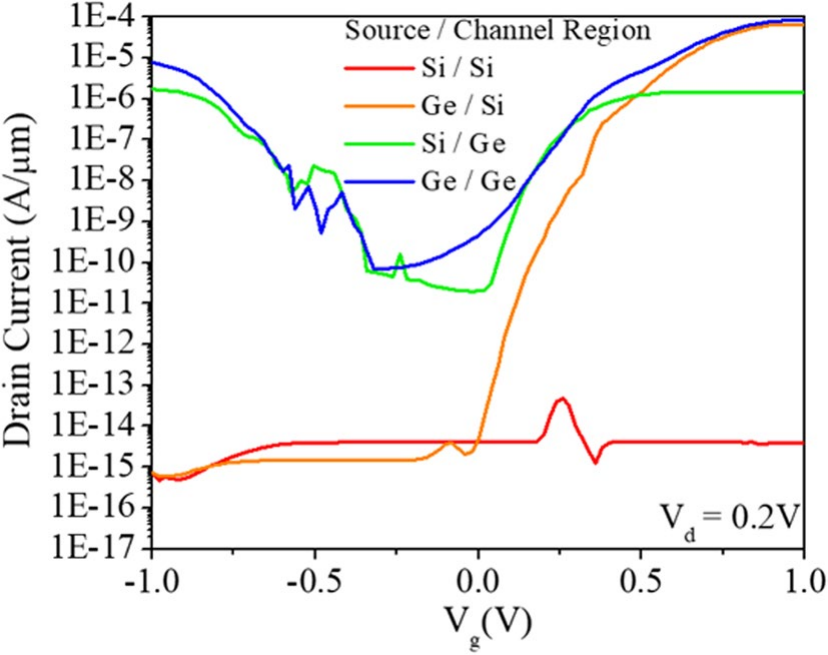
Transfer characteristics of GS-iTFET structure with different Source and Channel Regions materials at *V*_ds_ = 0.2 V

Traditional tunnel field effect transistors (TFETs) use single crystal silicon as the body material. However, germanium, which has a narrower bandgap than silicon, offers the potential for higher drive currents. However, the use of pure germanium as the body material leads to a significant increase in leakage current, while pure silicon exhibits limited tunneling due to its wider bandgap. To overcome this challenge, a hybrid approach is proposed where germanium is used in the source region while the channel region is replaced by silicon. This heterojunction configuration takes advantage of the band offset at the interface, resulting in an increased tunneling area between the source and channel in the on-state. Conversely, the wider bandgap of the silicon in the off-state makes tunneling less likely, ensuring low leakage current. This design strategy allows high drive currents to be achieved while maintaining low leakage current [21, 22]. Figure 6 illustrates the superior performance of the device in terms of high I_on_/I_off_ ratio at low voltage conditions.

### Effectively prevents point tunneling

**Fig. 7:**
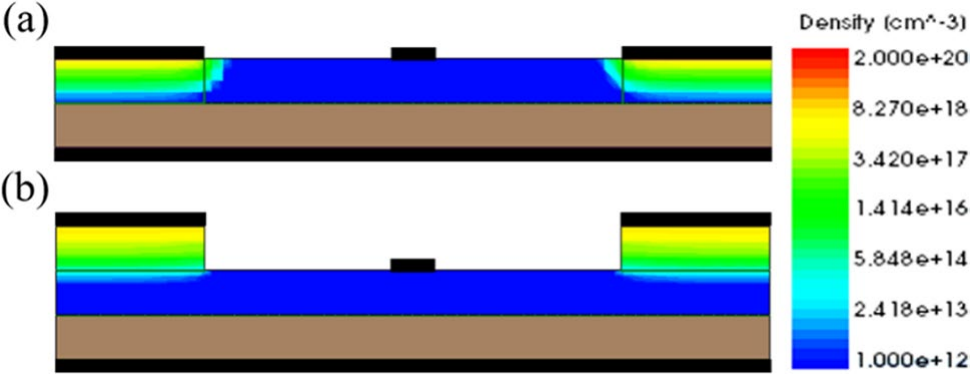
Comparison of hDensity with and without ETL in OFF State at *V*_gs_ = 1 V. **a** GS-iTFET and **b** ETL GS-iTFET

**Fig. 8:**
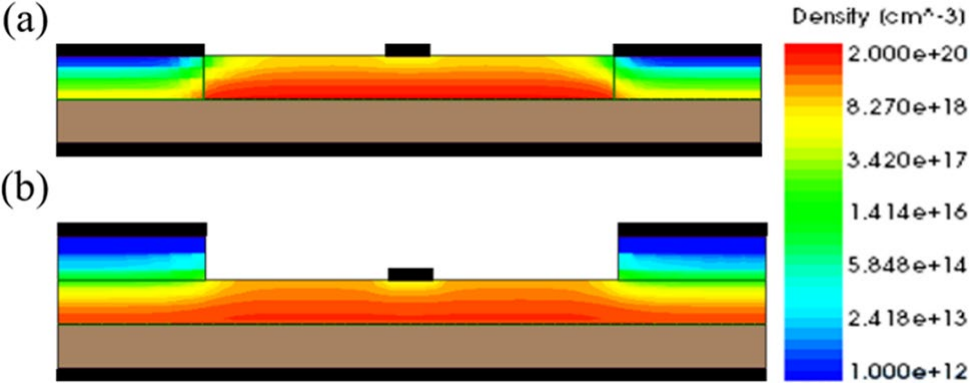
Comparison of eDensity with and without ETL in OFF State at *V*_gs_ = 1 V. **a** GS-iTFET and **b** ETL GS-iTFET

**Fig. 9:**
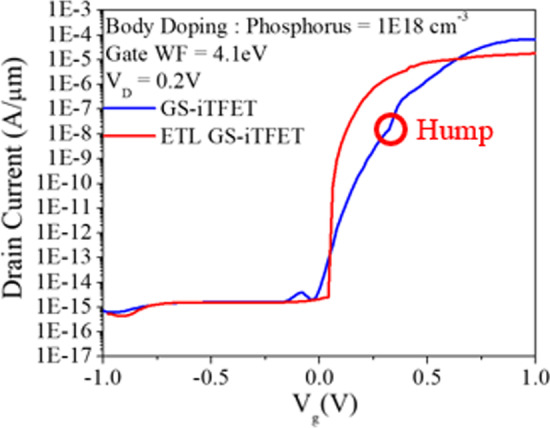
Transfer characteristics with and without ETL

**Fig. 10:**
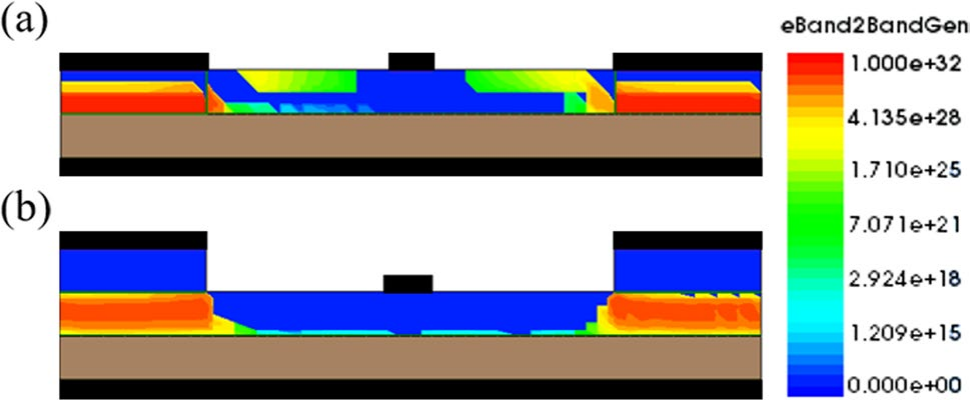
Comparison of eBTB Generation with and without ETL at *V*_gs_ = 1 V. **a** GS-iTFET and **b** ETL GS-iTFET

In our previous work, the GS-iTFET showed remarkable performance. However, the Schottky contact at the source region induced channel inversion, as shown in Fig. 7a. In addition, applying a positive gate bias resulted in a dense electron accumulation layer on the oxide surface, as shown in Fig. 8. Unfortunately, this configuration led to perpendicular tunneling between the source region and the electron accumulation layer, resulting in a hump as shown in Fig. 9. To overcome this challenge, we introduced an epitaxial tunneling layer (ETL) beneath the germanium source region. The addition of the ETL in Fig. 7b clearly separated the P-type region from the channel region, allowing tunneling only in the parallel direction with respect to the gate electric field. Comparative analysis in Fig. 10a showed a significant increase in lateral point tunneling in the absence of ETL. In contrast, Fig. 10b shows that the presence of ETL effectively suppresses point tunneling while maintaining line tunneling. It should be noted that after successful suppression of point tunneling, the conduction current naturally decreases. Although the on-state current does not retain its previous excellent performance, it exhibits a significantly steep behavior in the subthreshold region. Table 2 shows the *SS*_avg_ and *I*_on_/*I*_off_ ratio for both structures. It can be seen that the *SS*_avg_ of the ETL GS-iTFET improved from 74.4 (mV/dec) to 30.6 (mV/dec). However, the conduction current (*I*_on_) is slightly decreased from 6.54 × 10^–5^ (A/μm) to 1.77 × 10^–5^ (A/μm).

**Table 2:** Comparison of two structures

	GS-iTFET	ETL GS-iTFET
V_D_ (V)	0.2	0.2
*SS*_avg_ (mV/dec)	74.4	30.6
*I*_on_ (A/μm)	6.54 × 10^–5^	1.77 × 10^–5^
*I*_off_ (A/μm)	2.3 × 10^–15^	2.58 × 10^–15^
*I*_on_/*I*_off_ Ratio	2.84 × 10^10^	6.88 × 10^9^

### Using charge-enhancement insulation to depinning the Fermi level

**Fig. 11:**
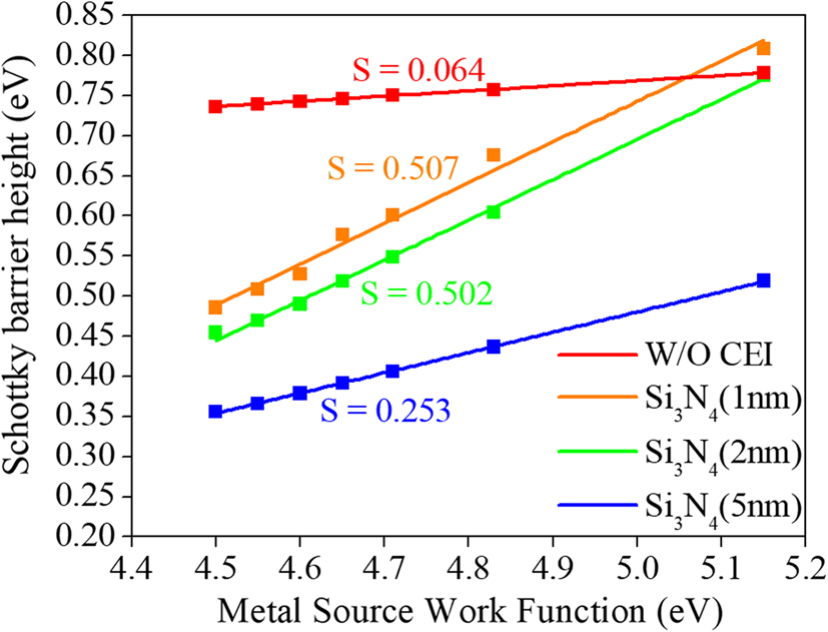
Extracted effective Schottky barrier heights for contacts without CEI, with 1 nm, 2 nm, and 5 nm Si_3_N_4_ layers. The S-factors for each case are also shown in the figure

In our previous work, we induced minority carrier holes to invert into P-type region in the N-type germanium source using a Schottky contact. However, direct metal contact on a semiconductor can cause Fermi level pinning effect [23], where the Schottky barrier is fixed. Since our device uses Schottky contact instead of ion implantation to create P-type region, fixing the Schottky barrier will limit our device's performance. Therefore, we need to insert a thin insulating layer between the metal and the semiconductor to mitigate the Metal-Induced Gap States (MIGS) effect and achieve Fermi level depinning. We added a 2 nm Si_3_N_4_ layer at the metal–semiconductor interface as the Charge-Enhancement Insulation (CEI), as shown in Fig. 1(c). The variations in Schottky barrier height at the metal–semiconductor interface are extracted for the cases without CEI and with Si_3_N_4_ layers of 1 nm, 2 nm, and 5 nm, respectively. The pinning factors S are obtained by calculating the slope of the linear regression lines [24]. From Fig. 11, it can be observed that the pinning factor S for the case without Si_3_N_4_ layer is 0.064, whereas for the case with a 1 nm Si_3_N_4_ layer, the pinning factor S is 0.507. The pinning factor S with CEI is higher than that without CEI, indicating that CEI can effectively alleviate the Fermi level pinning effect. Notably, the pinning factors for Si_3_N_4_ layers of 2 nm and 5 nm thickness are 0.502 and 0.253, respectively, both smaller than that for the 1 nm layer, suggesting that a thicker CEI does not necessarily lead to a better depinning effect.

**Fig. 12:**
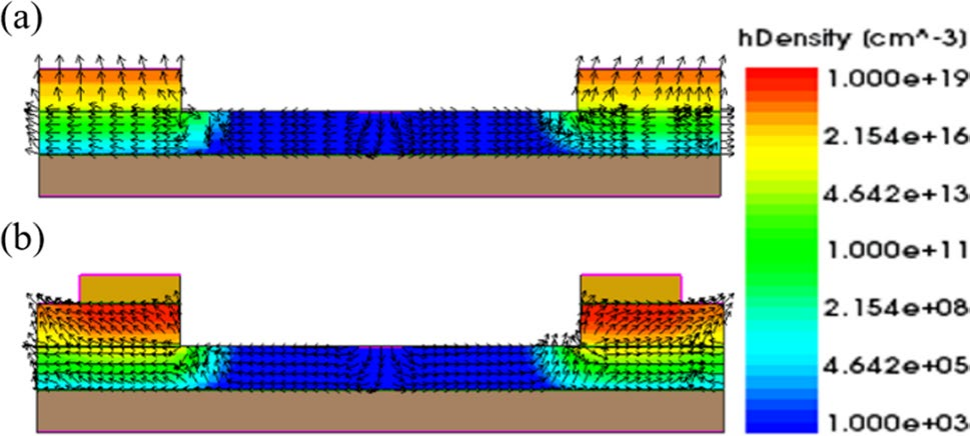
Direction of hole flow with and without the CEI for **a** W/O CEI and **b** With CEI at *V*_gs_ = 1 V

**Fig. 13:**
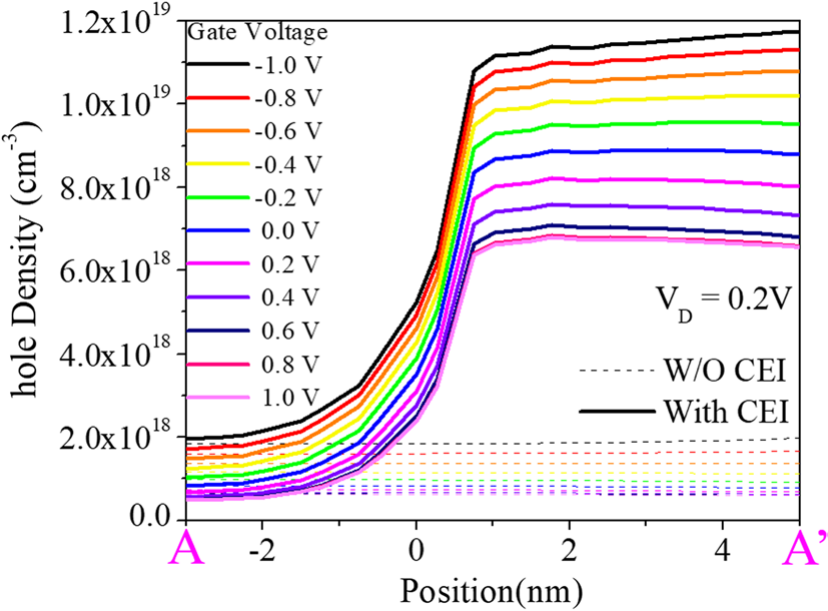
Electron concentration diagrams for the cutting line A to A' are shown for with and without the CEI at various gate voltages

**Fig. 14:**
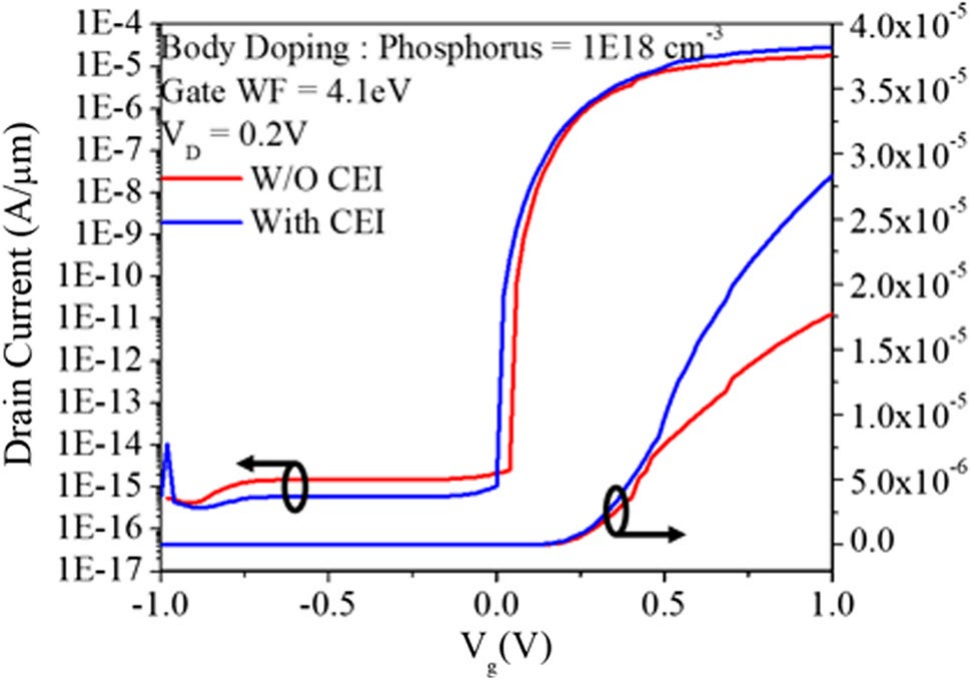
Transfer characteristics with and without the CEI are shown

Previously, we reduced the Fermi level pinning effect by adding a 2 nm Si_3_N_4_ layer at the metal–semiconductor interface. In addition to reducing the FLP effect, the Si_3_N_4_ layer can also reduce the loss of induced minority carrier holes from the metal. As shown in Fig. 12a, b, without an insulating layer, the holes flow out to the source metal in large quantities, resulting in the loss of induced minority carrier holes. On the other hand, with an insulating layer, the loss of holes is reduced, leaving more holes, as shown in Fig. 13. In addition, the increased concentration of induced carriers increases the conduction current, as shown in Fig. 14.

**Fig. 15:**
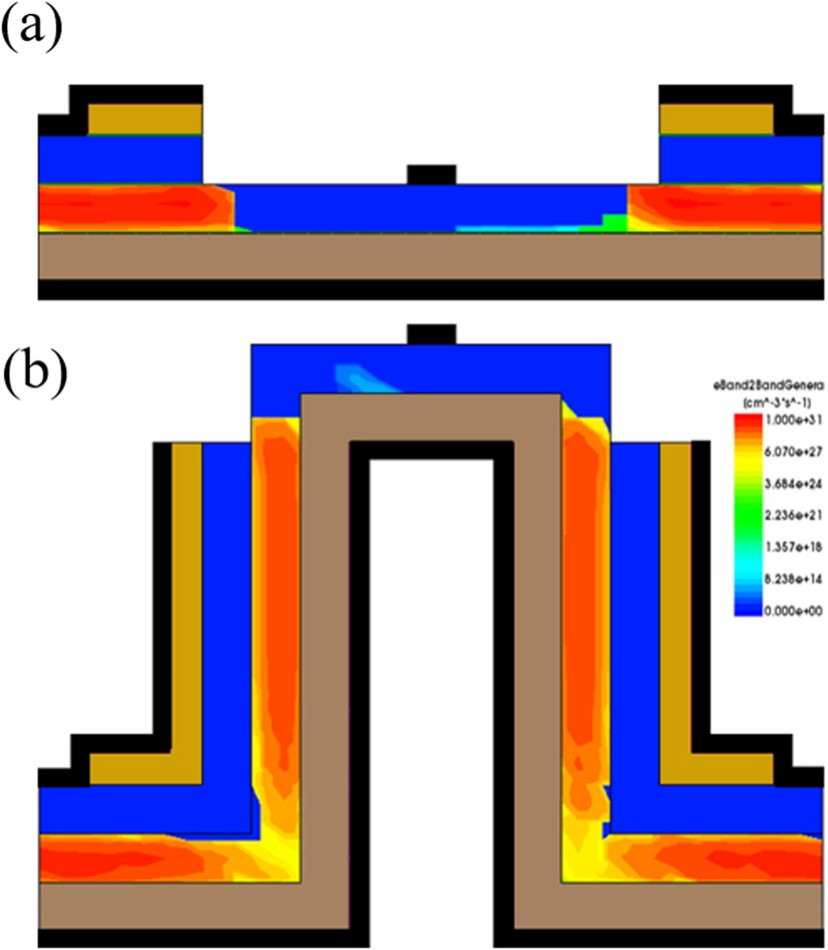
Comparison of eBTB Generation at different overlap lengths. **a** Overlap length 16 nm and **b** overlap length 58 nm

**Fig. 16:**
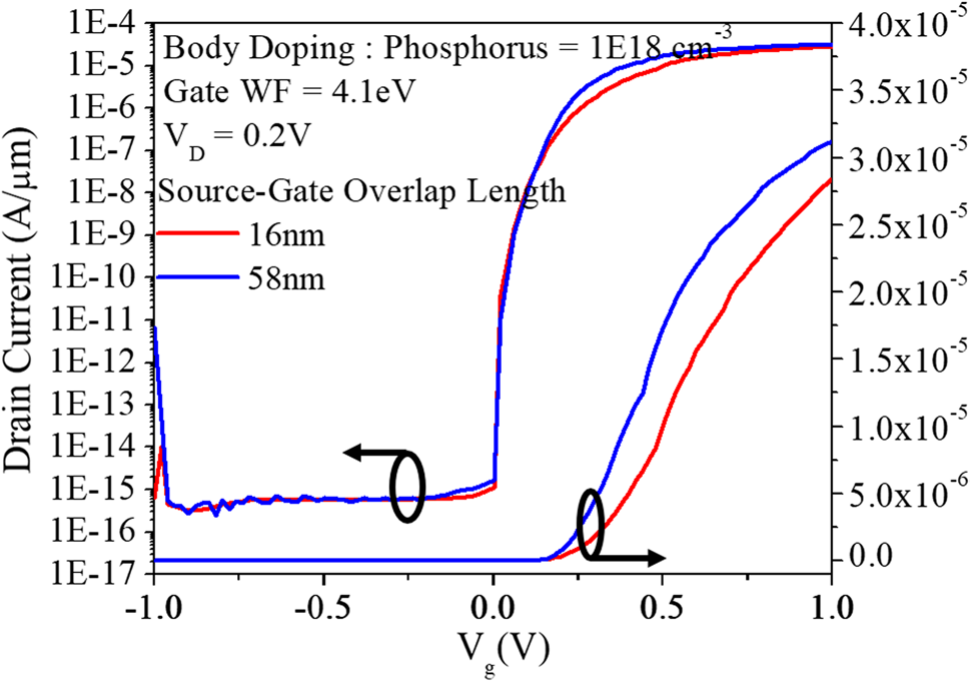
Transfer characteristics at different source-gate overlap lengths

Figure 15 shows that increasing the source-drain overlap length leads to a greater occurrence of tunneling and consequently increases the on-current as shown in Fig. 16.

**Fig. 17:**
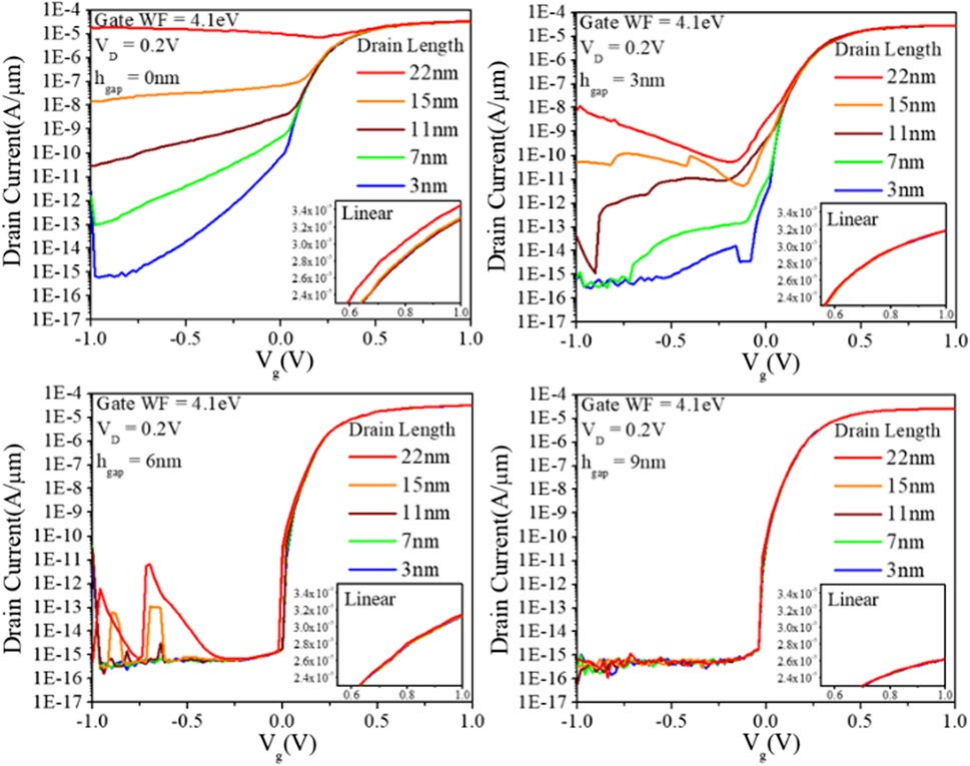
Transfer characteristics of the CEI ETL GS-iTFET. They are analyzed with respect to the modulation of *L*_D_ at different *h*_gap_ values

The distance between the source and drain is a critical factor affecting the performance of the device. In this study we investigate the effect of the gap height (*h*_gap_) between the source and the top surface and the length (*L*_D_) of the drain metal. Figure 17 shows the relationship between *L*_D_ and leakage current for different h_gap_ values. It can be seen that a shorter *h*_gap_ results in a higher leakage current as *L*_D_ increases. Conversely, increasing *h*_gap_ reduces the leakage current. This phenomenon can be attributed to direct tunneling from the source to the drain when the gap between them is too small, resulting in significant leakage. To ensure optimum electrical characteristics, increasing the *h*_gap_ allows a longer *L*_D_, but at the cost of reduced on-current due to a smaller tunneling area. In addition, the Gate-Induced Drain Leakage (GIDL) effect should be considered when extending the *L*_D_. For our device simulation, we set *h*_gap_ to 6 nm and *L*_D_ to 3 nm.

### Consideration of practical process defects

**Fig. 18:**
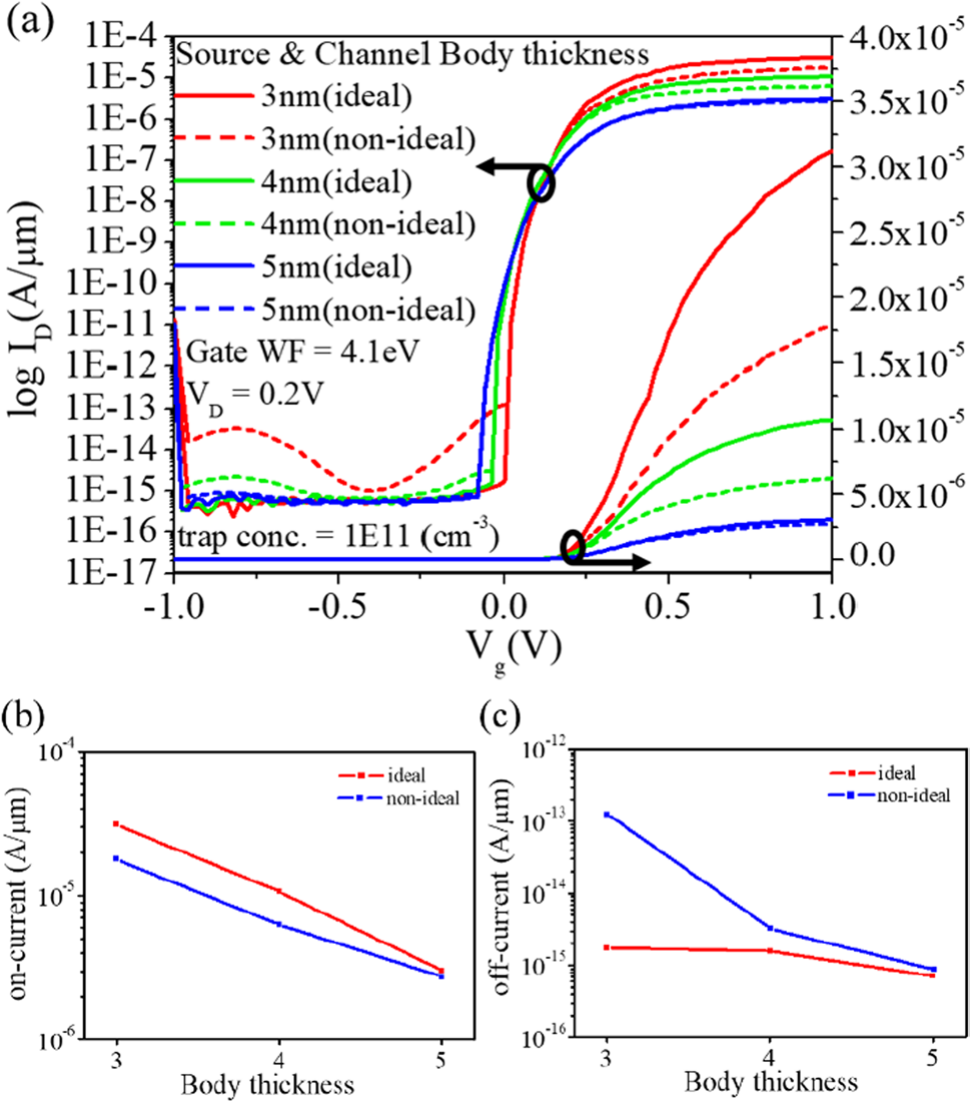
Ideal and non-ideal CEI ETL GS-iTFET with different body thicknesses. **a** Transfer characteristics, **b** on-current, **c** off-current

During the actual manufacturing process, repeated high-voltage stress can induce defects at interfaces and between different materials. These defects are located in the forbidden energy band between the conduction and valence bands and have the ability to trap carriers, resulting in a two-stage tunneling process that resembles a dormancy point. As a result, the required tunneling barrier is reduced, allowing carriers to more easily pass through the defects from the valence band to the conduction band, thereby increasing the device current. This phenomenon is commonly referred to as trap-assisted tunneling (TAT). However, the effect of TAT on the driving current is limited, while it significantly increases the leakage current. In addition, as device dimensions decrease and the electron mobility within the channel decreases, the on current decreases, as shown in Fig. 18.

**Fig. 19:**
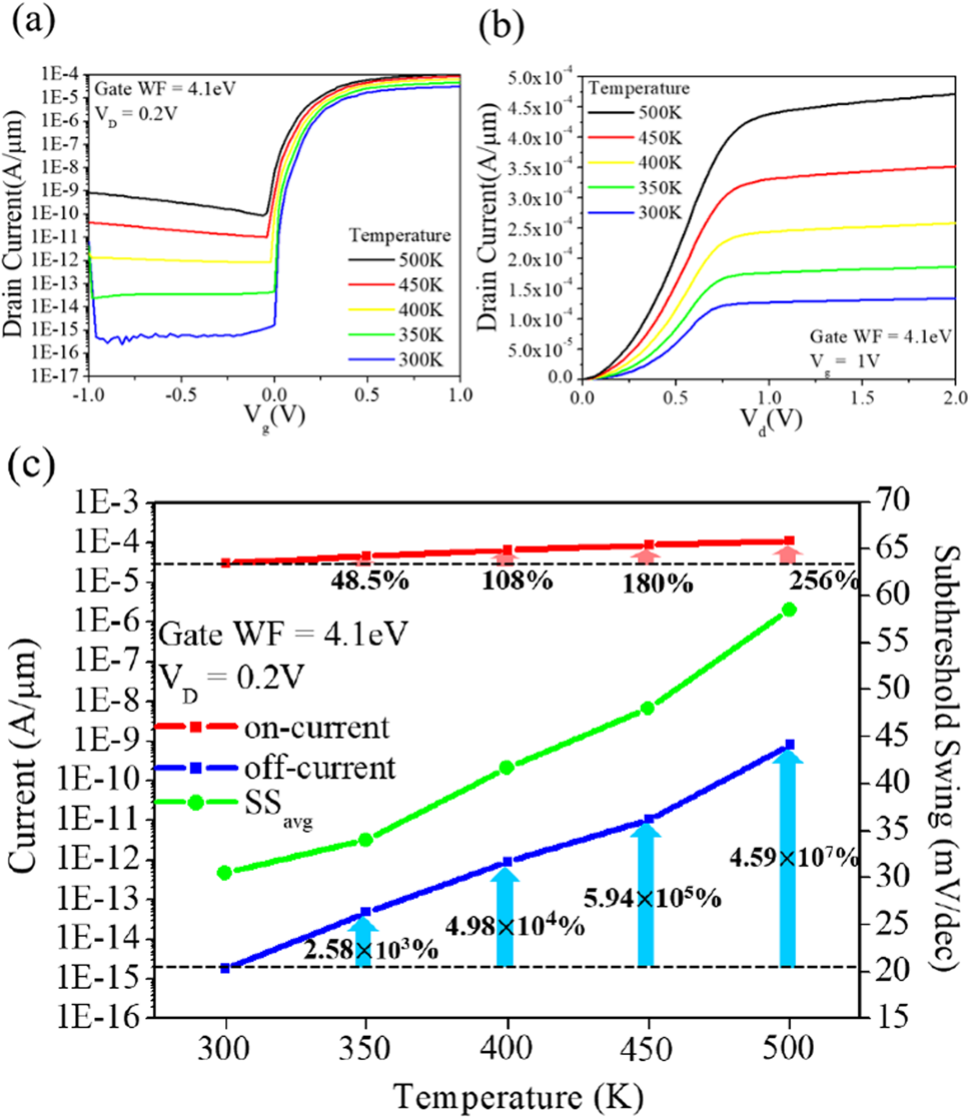
Temperature modulation of CEI ETL GS-iTFET for **a** *I*_d_–*V*_g_ curve, **b** *I*_d_–*V*_d_ curve, and **c** *I*_on_, *I*_off_ current

Temperature is a critical parameter that significantly affects the performance of semiconductor devices. As temperature increases, the bandgap of TFET devices decreases, leading to an increase in the band-to-band tunneling current [25]. However, the effect of temperature on the on-state current is not significant, whereas the off-state current is significantly affected by temperature, as shown in Fig. 19a. This difference is due to the fact that the off-state current is composed of trap-assisted tunneling (TAT) current, band-to-band tunneling (BTBT) current and Shockley–Read–Hall (SRH) recombination current, while the on-state current is mainly composed of BTBT current [26, 27]. From Fig. 19b, it can be observed that the TFET does not exhibit self-heating effects leading to a decrease in current even at high temperatures (500 K). Finally, we present the extracted data of the on-state current, off-state current and average subthreshold swing in Fig. 19c.

**Fig. 20:**
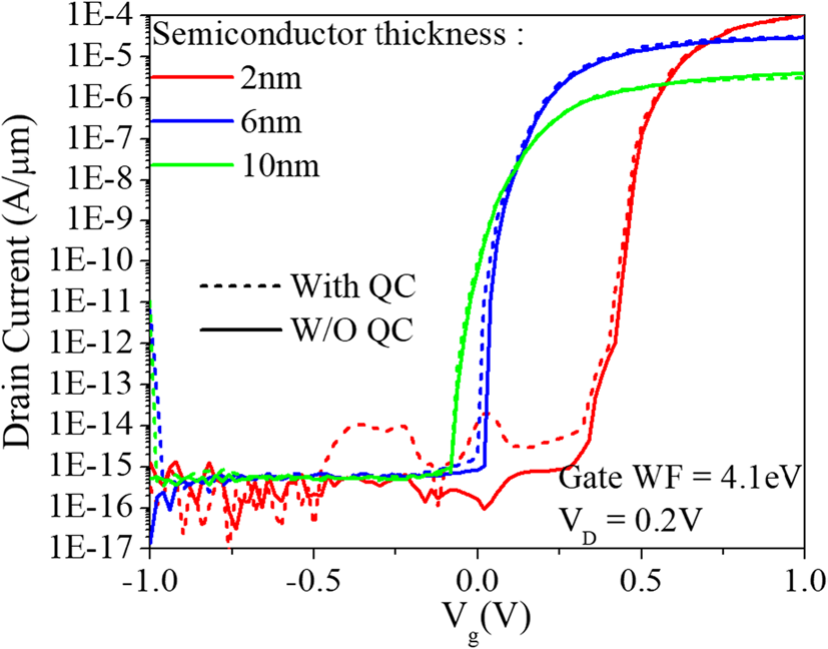
Simulated transfer characteristics of the CEI ETL GS-iTFET with and without Quantum Confinement (Density Gradient) considered

Due to the extremely small size of our device, we have considered the Quantum Confinement (QC) effect using the quantum momentum model (Density Gradient) [40]. Figure 20 shows the transfer characteristics with and without the use of QC at different semiconductor thicknesses. It is clear that the thinner the semiconductor thickness, the greater the effect.

**Fig. 21:**
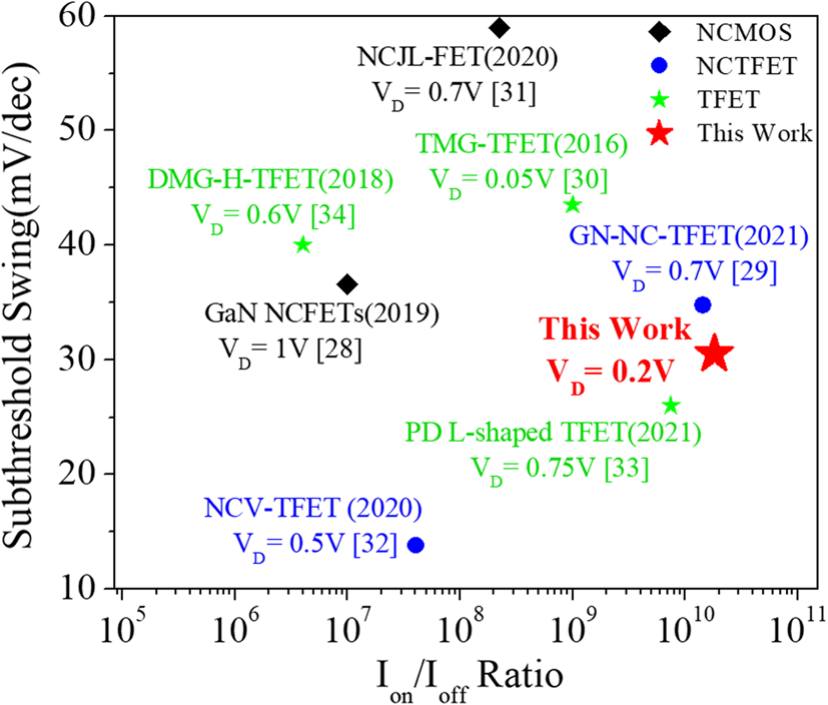
Performance comparison of devices. *SS*_avg_ of TFETs as a function of *I*_on_/*I*_off_

Finally, we conducted an evaluation of our proposed device's performance based on *I*_on_/*I*_off_ Ratio and SS, as illustrated in Fig. 21 [28–34]. Compared to other devices, the ETL GS-iTFET exhibited a remarkable *I*_on_/*I*_off_ Ratio. However, we also had to take into account the issue of FLP. Therefore, while mitigating FLP, the CEI ETL GS-iTFET achieved a slightly better  average subthreshold swing compared to the ETL GS-iTFET. It is worth noting that our simulations were performed without ion implantation, which resulted in an excellent performance while significantly reducing the thermal budget. Ultimately, the CEI ETL GS-iTFET demonstrates tremendous potential as one of the most promising candidates for the next generation of low-power devices.

### Conclusion

In this paper, we propose a CEI ETL GS-iTFET structure that uses a Schottky contact instead of ion implantation to induce minority carrier holes in the N-type body, thereby inverting the P-type region. Unlike conventional TFET devices that rely on point tunneling, our device utilizes source-gate overlap to create a line tunneling structure, resulting in a higher on-current. In addition, germanium material is incorporated as the source region to further increase the on-current, while an epitaxial tunnel layer (ETL) serves as the primary tunneling region to reduce the leakage current resulting from point tunneling between the source and drain. To mitigate Fermi level pinning (FLP), a layer of Si_3_N_4_ is introduced as charge enhancement isolation (CEI) between the source region and the Schottky metal. This layer effectively minimizes the loss of minority carrier holes and alleviates FLP effects, ultimately improving the efficiency and stability of the device. We also perform simulations to investigate non-ideal effects encountered during the manufacturing process and their impact on device performance. Under ideal conditions, the CEI ETL GS-iTFET exhibits exceptional performance with an average subthreshold swing (*SS*_avg_) of 30.5 mV/dec and an *I*_on_/*I*_off_ ratio of 1.81 × 10^10^. In summary, our proposed CEI ETL GS-iTFET has great potential to expand the application range of TFT technology and drive future advances in the development of thin film transistors (TFTs) for low power, low voltage and fast switching applications. This technological advancement addresses the demand for high efficiency, superior performance and low power consumption, ultimately improving overall device performance, stability and widespread adoption in future electronic devices.

## Data Availability

All the data are available from the corresponding author on reasonable request.
